# Trajectories of Sleep Disturbance and Self-Management of Chronic Conditions during COVID-19 among Middle-aged and Older Adults

**DOI:** 10.21203/rs.3.rs-2440390/v1

**Published:** 2023-01-10

**Authors:** Minjee Kim, Lauren Opsasnick, Stephanie Batio, Julia Y. Benavente, Morgan Bonham, Pauline Zheng, Rebecca M. Lovett, Stacy C. Bailey, Mary Kwasny, Daniela P. Ladner, Sherry HY. Chou, Jeffrey A. Linder, Sandra Weintraub, Yuan Luo, Phyllis C. Zee, Michael S. Wolf

**Affiliations:** Northwestern University; Northwestern University; Northwestern University; Northwestern University; Northwestern University; Northwestern University; Northwestern University; Northwestern University; Northwestern University; Northwestern University Transplant Outcomes Research Collaborative (NUTORC), Northwestern University; Northwestern University; Northwestern University; Northwestern University; Northwestern University; Northwestern University; Northwestern University

**Keywords:** COVID-19, sleep disturbance, self-management, subjective cognitive decline, medication adherence

## Abstract

**Background:**

The COVID-19 pandemic has had a widespread impact on sleep quality, yet little is known about the prevalence of sleep disturbance and its impact on self-management of chronic conditions during the ongoing pandemic.

**Objective:**

To evaluate trajectories of sleep disturbance, and their associations with one’s capacity to self-manage chronic conditions.

**Design:**

A longitudinal cohort study linked to 3 active clinical trials and 2 cohort studies with 5 time points of sleep data collection (July 15, 2020 – May 23, 2022).

**Participants:**

Adults living with chronic conditions who completed sleep questionnaires for two or more time points.

**Exposure:**

Trajectories of self-reported sleep disturbance across 5 time points.

**Main Outcomes:**

3 self-reported measures of self-management capacity, including subjective cognitive decline, medication adherence, and self-efficacy for managing chronic disease.

**Results:**

549 adults aged 23 to 91 years were included in the analysis. Two thirds had 3 or more chronic conditions; 42.4% of participants followed a trajectory of moderate or high likelihood of persistent sleep disturbance across the study period. Moderate or high likelihood of sleep disturbance was associated with older age (RR 1.57, 95% CI 1.09, 2.26, *P*<.05), persistent stress (RR 1.54, 95% CI 1.16, 2.06, *P*=.003), poorer physical function (RR 1.57, 95% CI 1.17, 2.13, *P*=.003), greater anxiety (RR 1.40, 95% CI 1.04, 1.87, *P*=.03) and depression (RR 1.63, 95% CI 1.20, 2.22, *P*=.002). Moderate or high likelihood of sleep disturbance was also independently associated with subjective cognitive decline, poorer medication adherence, and worse self-efficacy for managing chronic diseases (all *P*<.001).

**Conclusions:**

Persistent sleep disturbance during the pandemic may be an important risk factor for inadequate chronic disease self-management and potentially poor health outcomes in adults living with chronic conditions. Public health and health system strategies might consider monitoring sleep quality in adults with chronic conditions to optimize health outcomes.

## Introduction

The COVID-19 pandemic led to unprecedented disruptions to nearly every facet of daily life, with direct and indirect impacts on adults with chronic conditions. In addition to being at increased risk of severe illness from COVID-19,^1^ social distancing recommendations, economic hardships, and changes in healthcare access have created new challenges for these individuals in terms of effectively participating in the management of their own health.^2–6^ Beyond the direct effects of COVID-19, studies have suggested that the pandemic has made it more difficult to engage in requisite self-care behaviors, such as maintaining a healthy lifestyle and taking prescribed medication. This may be the result of many factors, including more infrequent engagement with healthcare professionals and care teams, disruptions in daily routine, increased social isolation, loneliness and/or stress resulting in depression and anxiety, as well as subsequent cognitive symptoms including difficulties in memory, attention, and information processing that can readily affect one’s health literacy skills and treatment adherence.

Changes in sleep quality might also have formidable consequences to an adult’s capacity to self-manage chronic conditions. Early in the pandemic, a significant increase in sleep disturbance was reported among older adults with chronic conditions,^7^ colloquially labeled as the “coronasomnia” phenomenon.^8^ Disturbed sleep has previously been associated with reduced self-management behaviors, missed medical appointments, and worse chronic disease outcomes.^9, 10^ Yet little is known about how sleep quality changed during the pandemic in adults living with chronic conditions and whether certain sleep trajectories have affected their ability to effectively manage their health.

Leveraging an ongoing, NIH-sponsored COVID-19 & Chronic Conditions (C3) study, we assessed the prevalence of persistent sleep disturbance across the first two years of the pandemic, and sought to investigate whether prolonged disturbed sleep was associated with a compromised capacity to self-manage chronic conditions. As a longitudinal cohort study, adults living with one or multiple chronic conditions have been interviewed 8 times to date since the beginning of the pandemic, of which sleep quality was examined at 5 of these assessments. We specifically sought to examine trajectories of sleep disturbance between July 2020 and May 2022, and to investigate associations between sleep disturbance trajectories and self-management capacity. Findings therefore might help reveal those individuals at greater risk of experiencing persistent sleep disturbance, and further inform future public health or health system strategies for screening and intervention to optimize health outcomes.

## Methods

This study follows the Strengthening the Reporting of Observational Studies in Epidemiology reporting guidelines. The study was approved by the Northwestern University Institutional Review Board.

### Study Design

The C3 study is an ongoing, telephone-based survey that began at the onset of the COVID-19 pandemic in the U.S among adults with chronic conditions. The initial survey was conducted from March 13 to 20, 2020 during the first week of the outbreak in Chicago, Illinois. Over the course of two years, beginning in March 27, 2020 through May 23, 2022, seven subsequent study interviews, referred to as waves, were conducted (See **Supplement – Table S1**).

### Study Participants

Eligibility criteria included participants actively enrolled in one of five ongoing, National Institutes of Health (NIH)-sponsored health services research projects. All parent studies excluded individuals with severe hearing, vision, or cognitive impairments. Four of the five research studies included only English-speaking subjects and one study included English- and Spanish-speaking subjects. The five parent studies have been published elsewhere.^11–14^ Across all five parent studies, participants are comprised of mostly middle-aged or older adults with multiple chronic conditions. All participants are receiving medical care at one of five academic internal medicine practices or two federally qualified health centers throughout the greater Chicago metropolitan area.

Trained research staff recruited participants from their parent studies to participate in a telephone survey pertaining to COVID-19. Survey data were collected using REDCap. Each survey averaged 20–40 minutes in duration, and participants were compensated with a $10 to $15 gift card for their time. A total of 672 participants were enrolled in the study and completed a Wave 1 interview. Cooperation rates at follow-up interviews has continued to be high, ranging from 72 to 93%. To focus on sleep trajectories during the COVID-19 pandemic, we limited the current study to 549 participants who provided sleep data from at least two Waves (See **Supplement – Table S2**).

### Exposure: Assessment of Sleep Disturbance

From Waves 4 through 8, self-reported sleep quality was measured using the Patient-Reported Outcomes Information System 4-item short-form battery for sleep disturbance (PROMIS-SD). PROMIS-SD items assess perceived difficulties and concerns with falling asleep and staying asleep, and perceptions of the adequacy of sleep. Higher scores represent poorer sleep quality. Presence of sleep disturbance was conservatively defined as PROMIS-SD T-score > 55, using a developer-recommended threshold of 0.5 standard deviation from the population mean.^15, 16^ The PROMIS-SD T-score has also been calibrated against Pittsburgh Sleep Quality Index (PSQI)^17^, another commonly used measure of sleep quality.

A PROMIS-SD T-score > 55 is equivalent to a PSQI score of > 10.^18^ In contrast, the most commonly used PSQI cutoff to define sleep disturbance is PSQI > 5, equivalent to PROMIS-SD T-score > 45.^18–23^ Due to this discrepancy, we conducted sensitivity analyses using T-score threshold > 45 to define sleep disturbance to facilitate comparison with other studies.

### Outcomes: Assessment of Self-Management Ability

The primary outcomes of interest were measures of self-management, using the latest available data for each participant. Subjective cognitive decline was measured using a subset of items from the *Everyday Cognition* (ECog) scale, a validated, self-reported measure of cognitive functioning.^24, 25^ ECog scores range from 1 to 4, with higher scores indicating worse cognitive abilities.^24^ Next, medication adherence was measured using the Ask-12 medication survey.^26^ Ask-12 scores range from 12–60, with higher scores indicating poorer adherence.^27^ This tool has demonstrated good internal consistency reliability (α = 0.75) and test-retest reliability (intraclass correlation 0.79) in prior studies; convergent validity was also demonstrated with pharmacy claims data.^27^ Finally, self-efficacy was measured using the Lorig’s Self-Efficacy for Managing Chronic Diseases 6-item Scale. This tool covers multiple domains of chronic disease self-management, including symptom control, role function, emotional functioning and communicating with physicians. Scores range from 1–10 and lower scores indicate lower self-efficacy.^28^

### Covariates: Demographic Characteristics, Physical and Mental Health

Across all five NIH parent studies, there was prior, uniform collection of participant demographics (age, sex, race and ethnicity), socioeconomic status (household income, educational attainment) and self-reported chronic conditions. All studies included a measure of health literacy, and participants were classified as having low, marginal, or adequate health literacy, as previously described in detail.^11^

The Cohen 10-item Perceived Stress Scale (PSS), adapted to respond to perceived stress in response to COVID-19, was used to measure the perception of stress from Waves 4 through 8.^29^ From Waves 3 through 8, mental health was measured using PROMIS short-form batteries for depression and anxiety where clinically significant symptoms of depression and anxiety were defined by T-score > 55.^16, 30^ Physical health was measured at Waves 5 and 7 using PROMIS 10-item short-form battery for physical function where low physical function was defined by T-score < 45.^15, 16, 31, 32^ The earliest and latest available data for PROMIS physical function, depression, anxiety, and PSS were used to define the presence or absence of persistently low physical function, persistent depression, persistent anxiety, and persistent stress, respectively.

### Statistical Analysis

We identified groups of individuals following similar progressions of sleep disturbance over multiple waves and classified them into trajectory groups using the *traj* command in Stata/SE, version 15 (StataCorp, College Station, TX, US).^33^ This method estimates discrete mixture models on longitudinal data, in our case assuming a Bernoulli distribution for the dichotomous sleep disturbance variable. We used the Bayesian information criterion to determine the number of discrete trajectories in the data. Participants were assigned to a trajectory based on posterior probabilities of belonging to each group.^34^

Next, we completed a series of analyses to understand (1) which participant characteristics were associated with distinct trajectories of sleep disturbance and (2) whether sleep disturbance trajectories were subsequently associated with measures of self-management abilities. First, descriptive statistics were calculated for all participant characteristics and survey responses. Associations of sleep disturbance trajectories with participant characteristics and outcome measures were examined in bivariate analyses using chi-square tests, t-tests, or one-way ANOVA tests, as appropriate. Multivariable Poisson models were used to estimate relative risks (RR; with 95% confidence intervals (CIs)) of following a certain trajectory.^35^ All multivariable models included sex, parent study, and variables that were significantly associated with sleep disturbance trajectory in bivariate analyses. Given overlap between similar, but distinct, constructs related to physical and mental health and concern for overadjustment, in each model we included only one of the measures of physical or mental health that were significantly associated with sleep disturbance trajectory in the bivariate analyses.

For outcome measures significantly associated with sleep disturbance trajectory in bivariate analyses, multiple regressions were used to estimate least square means (LSM; with 95% CIs). All models included a *priori* covariates of sex, parent study as well as age, race/ethnicity, poverty, and education, based on their established associations with self-management abilities.^25, 36^

We conducted a sensitivity analysis comparing baseline characteristics of C3 study participants included in this analysis from those who were excluded. All statistical analyses were performed using Stata/SE, version 15 (StataCorp).^33^ We considered *P* < .05 (2-sided) to be significant.

## Results

### Sleep Disturbance Trajectories and Study Sample Characteristics

Of the 549 participants with sleep measures for at least two timepoints between July 15, 2020 and May 23, 2022, ages ranged between 23 and 91 years (mean [SD], 63 [11]); nearly two thirds were women and 45.2% were White, with a third (29.3%) Black and 20.6% Hispanic/Latino (H/L) (Table 1). Participants included in this study were not significantly different from those who were excluded (N = 123) regarding age, sex, race/ethnicity, income, education, health literacy, or the number of chronic conditions (**Supplement – Table S3**).

**Figure 1:**
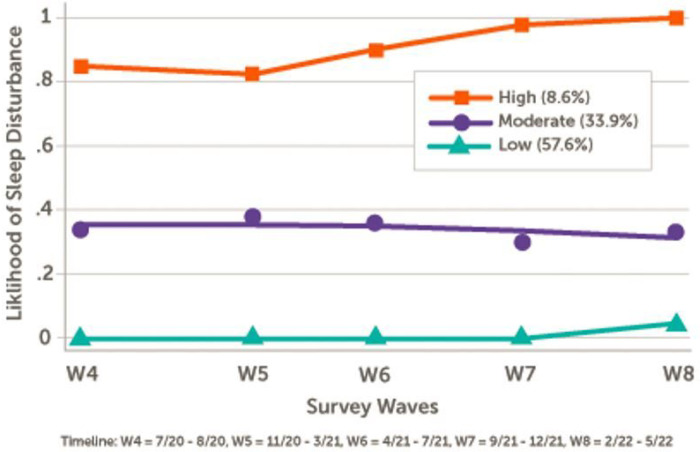
Trajectory of Sleep Disturbance (Wave 4 to Wave 8) Between July 2020 and May 2022 and across five survey Waves, three distinct trajectories of likelihood of sleep disturbance were identified. Sleep disturbance at each survey Wave was defined by PROMIS Sleep Disturbance T-score >55.

Three trajectory groups of sleep disturbance were estimated using a PROMIS-SD > 55 cutoff and labeled as follows: (1) low sleep disturbance, including 57.6% of participants who were unlikely to experience sleep disturbance throughout the study period; (2) moderate sleep disturbance, including 33.9% of participants who maintained a moderate likelihood of experiencing sleep disturbance; and (3) high sleep disturbance, including 8.6% of participants who started at a high likelihood of sleep disturbance, which gradually worsened to nearly 100% likelihood by the end of the study period (Fig. 1). In bivariate analyses, age < 60 years, H/L, living below poverty level, persistent moderate-to-high stress, poorer physical function, and symptoms of depression and anxiety were associated with a high sleep disturbance trajectory (Table 1). In multivariable analyses, age < 60 (RR [95% CI] 1.57 [1.09, 2.26], *P* < .05) and persistent stress (1.54 [1.16, 2.06], *P* = .003), low physical function (1.57 [1.17, 2.13], *P* = .003), anxiety (1.40 [1.04, 1.87], *P* = .03), and depression (1.63 [1.20, 2.22], *P* = .002) were significantly associated with a moderate or high sleep disturbance (Table 2).

### Sleep Disturbance Trajectories and Self-management Outcomes

High likelihood of sleep disturbance was associated with subjective cognitive decline (mean [SD] ECog: 1.7 [0.6] vs low sleep disturbance, 1.3 [0.36]; *P* < .001), poorer medication adherence (25.3 [6.8] vs 19.3 [5.4]; *P* < .001), and lower self-efficacy for managing chronic diseases (6.1 [2.6] vs 8.2 [1.8]; *P* < .001; Table 1). In multivariable analyses, participants categorized to a moderate or high likelihood of sleep disturbance demonstrated subjective cognitive decline (ECog LSM [95% CI]: 1.51[1.44, 1.59] vs 1.32 [1.25, 1.39]; *P* < .001), poorer medication adherence (Ask-12: 22.2 [21.3, 23.2] vs 19.8 [19.0, 20.7]; *P* < .001), and lower self-efficacy for managing chronic diseases (7.0 [6.67, 7.33] vs 7.81 [7.51, 8.11], *P* < .001; Table 3), compared to those following a low sleep disturbance. In addition, older age was associated with subjective cognitive decline (ECog LSM [95% CI]: 1.48 [1.38, 1.58] for ≥ 70 vs 1.35 [1.26, 1.44] for ≤ 60; *P* = .002), while H/L was associated with poorer medication adherence (22.2 [20.8, 23.6] vs 21.4 [20.3, 22.5] for Non-H/L White; *P* = .001) and living below poverty level was associated with subjective cognitive decline and lower self-efficacy (ECog: 1.49 [1.40, 1.58] vs 1.35 [1.28, 1.41]; self-efficacy: 7.02 [6.62, 7.42] vs 7.79 [7.51, 8.07]; *P* < .01 for both; Table 3).

### Sensitivity Analysis

In the sensitivity analysis applying an alternative PROMIS SD T-score threshold of > 45 (i.e., equivalent to PSQI > 5) to define sleep disturbance at each Wave, three distinct trajectories of sleep disturbance were identified (**Supplement – Figure S1**). Nearly two thirds of the participants (63.5%) were categorized as having a high likelihood of persistent sleep disturbance over two years of the pandemic, while 26.8% were identified as having a moderate likelihood of sleep disturbance, which worsened during the second COVID-19 surge then gradually improved by the end of the study period. A few participants (9.7%) were categorized to the third trajectory where they started with a low likelihood of sleep disturbance which further improved throughout the year 2021 then slightly worsened after the Omicron surge. We achieved similar results regarding associations of a high likelihood of sleep disturbance trajectory with self-management outcomes, compared to the main analysis above using a PROMIS SD T-score threshold of > 55.

## Discussion

In this sample of U.S. adults living with chronic conditions who were surveyed throughout the beginning years of the pandemic, we identified three distinct trajectories of sleep disturbance. Over half of participants maintained a low likelihood of experiencing persistent poor sleep. However, nearly a half demonstrated a moderate to high likelihood of sleep disturbance. Those who were younger and in persistently poor physical and mental health were more likely to experience sleep disturbance. Those experiencing poor sleep were more likely to report problems with their cognition and self-management capacity, including inadequate medication adherence.

Self-management abilities are essential for any adult living with chronic conditions. Those who struggle to self-manage their health may be more vulnerable to adverse health outcomes during the ongoing pandemic where the level of support from family, community, and healthcare professionals has been variable and changes in healthcare delivery methods have made them less readily accessible.^4, 6, 37^ Poor self-management of chronic conditions has been reported even among patients who adapted to restructured healthcare services,^38^ which likely contributed to an estimated excess of 44,600 non-COVID-19 deaths that occurred in the U.S. from March through August 2020, with the most common causes being diabetes, dementia, and heart disease.^39^ These early pandemic data are concerning for a large number of people with chronic conditions; some may have struggled to monitor their symptoms or even avoided healthcare settings due to fear of contracting COVID-19. While this is not entirely clear, timely identification of individuals at risk of inadequate self-management and provision of appropriate support could be helpful in minimizing downstream consequences of chronic care disruption during the pandemic.

Leveraging a unique opportunity to examine a group of adults living with chronic conditions across two years of COVID-19 pandemic, we report persistent sleep disturbance as a potential risk factor for poorer self-management abilities. There may be several potential mechanisms linking sleep disturbance with self-management capacity. First, sleep disturbance can negatively affect cognitive performance, especially executive functioning.^40–45^ Self-management of chronic conditions involves complex tasks that require sustained attention and appropriate information processing on a daily basis, such as checking blood glucose and responding to results, following certain diets, engaging in regular physical activities, adhering to medication regimens, and monitoring symptoms and contacting healthcare professionals when necessary. Although we did not have objective cognitive assessments in this study, individuals with a moderate or high likelihood of persistent sleep disturbance reported greater subjective decline compared to those at a low likelihood of sleep disturbance. Second, sleep disturbance may reduce motivation to engage in health-promoting behaviors and lower confidence in one’s ability to manage one’s own health.^46–48^

Additionally, younger adults were more likely to experience sleep disturbance, similar to findings from a nationally representative sample.^49^ The pandemic has created unprecedented challenges for middle-aged adults who have had to juggle a myriad of roles including financial and caregiving responsibilities in the midst of school closures and economic hardship, while also managing their own chronic conditions. Future research should seek to examine long-term trends and consequences of sleep health in this age group.

Regarding other factors associated with self-management, H/L adults reported poorer medication adherence than their White counterparts and those living below poverty level were more likely to demonstrate subjective cognitive decline and worse self-efficacy. The COVID-19 pandemic has been disproportionately affecting chronic disease management among communities that are more socioeconomically disadvantaged, widening chronic disease disparities.^5^ Public health and health system interventions to extend self-management support for marginalized individuals and communities will be critical to mitigate pandemic-exacerbated health inequities.

This research has several limitations. Foremost, as this study was done in a subset of active participants in five ongoing research projects focused on adults with at least one chronic condition conducted in one large city in the U.S., the results may not be generalizable to other populations, especially those who are younger and without chronic health concerns. Second, sleep data were not collected in earlier waves of the longitudinal C3 study or as part of the parent studies, limiting our ability to make comparisons to pre-pandemic sleep quality. Third, objective measures of sleep, cognition, or physical function were not collected in this survey-based study. Fourth, attrition of the samples in later survey Waves may introduce bias. However, over 85% of participants who provided the initial sleep data remained in the study throughout the subsequent four Waves, and those who were excluded in this analysis were not significantly different from the analyzed sample. The strengths of this study include (1) longitudinal examination of higher risk adults who are under-represented in the existing literature yet are among the most vulnerable to the ongoing effects of the pandemic; (2) a socioeconomically and racially/ethnically diverse sample; and (3) detailed measures of self-management abilities using validated instruments.

## Conclusions

Persistent sleep disturbance during two years of COVID-19 pandemic was associated with poorer self-management abilities in adults living with chronic conditions. Health inequity in self-management during the pandemic was apparent. Public health and health system interventions should address sleep health in the ongoing management of chronic conditions, particularly in socioeconomically disadvantaged populations, to prevent morbidity and mortality associated with inadequate self-care.

## Figures and Tables

**Table 1:** Predictor and Outcome Variables, Overall and by Sleep Disturbance Trajectory

Variable, No (%)	Overall (N = 549)	Low Sleep Disturbance (n = 316)	Moderate Sleep Disturbance (n = 186)	High Sleep Disturbance (n = 47)	P-value
**Predictors**					
Age Group					
< 60	191 (34.8)	90 (28.5)	79 (42.5)	22 (46.8)	
60–69	204 (37.2)	126 (39.9)	56 (30.1)	22 (46.8)	**< 0.001**
≥ 70	154 (28.1)	100 (31.7)	51 (27.4)	3 (6.4)	
Gender					
Female	338 (61.6)	185 (58.5)	118 (63.4)	35 (74.5)	0.09
Male	211 (38.4)	131 (41.5)	68 (36.6)	12 (25.5)	
Race/ethnicity					
Hispanic/Latino	113 (20.6)	62 (19.6)	33 (17.7)	18 (38.3)	
Non-Hispanic/Latino White	248 (45.2)	156 (49.4)	80 (43.0)	12 (25.5)	**0.01**
Non-Hispanic/Latino Black	161 (29.3)	84 (26.6)	63 (33.9)	14 (29.8)	
Other	27 (4.9)	14 (4.4)	10 (5.4)	3 (6.4)	
Living below Poverty Level ^+^					
Yes	155 (28.4)	70 (22.3)	61 (33.0)	24 (51.1)	**< 0.001**
No	391 (71.6)	244 (77.7)	124 (67.0)	23 (48.9)	
Education					
High School Grad or Less	135 (24.6)	76 (24.1)	46 (24.7)	13 (27.6)	0.07
Some College	141 (25.7)	69 (21.8)	55 (29.6)	17 (36.2)	
College Degree or Higher	273 (49.7)	171 (54.1)	85 (45.7)	17 (36.2)	
Health Literacy					
Low	122 (22.2)	66 (20.9)	43 (23.1)	13 (27.7)	
Marginal	125 (22.8)	65 (20.6)	44 (23.7)	16 (34.0)	0.11
Adequate	302 (55.0)	185 (58.4)	99 (53.2)	18 (38.3)	
Number of Chronic Conditions					
1	120 (21.9)	66 (20.9)	39 (21.0)	15 (31.9)	
2	92 (16.8)	58 (18.4)	28 (15.0)	6 (12.8)	0.39
3 or more	337 (61.4)	192 (60.8)	119 (64.0)	26 (55.3)	
Persistent Stress ^+^					
Yes	149 (27.4)	59 (18.8)	59 (32.1)	31 (67.4)	**< 0.001**
No	395 (72.6)	255 (81.2)	125 (67.9)	15 (32.6)	
Persistently Low Physical Function ^+^					
Yes	201 (38.7)	89 (29.7)	81 (45.5)	31 (73.8)	**< 0.001**
No	319 (61.3)	211 (70.3)	97 (54.5)	11 (26.2)	
Persistent Anxiety ^+^					
Yes	138 (25.2)	55 (17.5)	56 (30.1)	27 (57.5)	**< 0.001**
No	410 (74.8)	260 (82.5)	130 (69.9)	20 (42.5)	
Persistent Depression					
Yes	107 (19.5)	34 (10.8)	47 (25.3)	26 (55.3)	**< 0.001**
No	442 (80.5)	282 (89.2)	139 (74.7)	21 (44.7)	
**Outcomes**					
ECog Score [1–4], M (SD)	1.4 (0.48)	1.3 (0.36)	1.5 (0.56)	1.7 (0.60)	**< 0.001**
ASK-12 Total [12–60], M (SD)	20.6 (6.1)	19.3 (5.4)	21.5 (6.2)	25.3 (6.8)	**< 0.001**
Lorig Self-Efficacy [1–10], M (SD)	7.8 (2.0)	8.2 (1.8)	7.5 (2.0)	6.1 (2.6)	**< 0.001**

+ Poverty had 3 participants missing data; Persistent stress had 5 participants missing data; Persistently low physical function had 55 participants missing data; Persistent anxiety had 1 participant missing data.

**Table 2:** Multivariate Analysis with Sleep Disturbance as Outcome of Interest

Variable	Moderate/High Sleep Disturbance	Moderate/High Sleep Disturbance	Moderate/High Sleep Disturbance	Moderate/High Sleep Disturbance	Moderate/High Sleep Disturbance
	RR (95% CI)	RR (95% CI)	RR (95% CI)	RR (95% CI)	RR (95% CI)
Age Group					
< 60	**1.57 (1.09, 2.26)** *	**1.63 (1.13, 2.35)** *	**1.59 (1.09, 2.32)** *	**1.57 (1.09, 2.27)** *	**1.53 (1.06, 2.21)** *
60–69	**1.13 (0.78, 1.64)** *	**1.12 (0.77, 1.63)** *	**1.12 (0.77, 1.64)** *	**1.14 (0.78, 1.65)** *	**1.12 (0.77, 1.62)** *
≥ 70	REF	-	-	-	-
Sex					
Male	REF	-	-	-	-
Female	1.16 (0.87, 1.56)	1.14 (0.85, 1.54)	1.07 (0.79, 1.45)	1.12 (0.83, 1.50)	1.11 (0.82, 1.49)
Race/ethnicity					
Hispanic/Latino	0.88 (0.60, 1.31)	0.84 (0.56, 1.24)	0.83 (0.55, 1.24)	0.86 (0.58, 1.27)	0.86 (0.58, 1.27)
Non-Hispanic/Latino White	REF	-	-		-
Non-Hispanic/Latino Black	1.11 (0.80, 1.53)	1.08 (0.78, 1.50)	0.99 (0.71, 1.40)	1.15 (0.82, 1.63)	1.11 (0.80, 1.54)
Living Below Poverty Level					
Yes	1.36 (0.99, 1.87)	1.25 (0.91, 1.73)	1.22 (0.88, 1.71)	1.30 (0.94, 1.79)	1.25 (0.91, 1.74)
No	REF	-	-	-	-
Education					
High School Grad or Less	1.01 (0.69, 1.47)	0.99 (0.68, 1.45)	1.05 (0.72, 1.54)	1.00 (0.69, 1.46)	0.99 (0.68, 1.45)
Some College	1.21 (0.86, 1.69)	1.17 (0.84, 1.65)	1.21 (0.85, 1.71)	1.15 (0.82, 1.63)	1.14 (0.81, 1.61)
College or higher	REF	-	-	-	-
Persistent Stress					
Yes	--	**1.54 (1.16, 2.06)** **	--	--	--
No	--	REF	--	--	--
Persistently Low Physical Function					
Yes	--	--	**1.57 (1.17, 2.13)** **	--	--
No	--	--	REF	--	--
Persistent Anxiety					
Yes	--	--	--	**1.40 (1.04, 1.87)** *	--
No	--	--	--	REF	--
Persistent Depression					
Yes	--	--	--	--	**1.63 (1.20, 2.22)** **
No	--	--	--	--	REF

RR, risk ratio; CI, confidence interval.

Significant associations are boldened

* = P-value < .05

** = P-value < .01

**Table 3:** Multivariate Analysis with Sleep Disturbance as Main Predictor of Interest

Variable	ECOG Score	ASK-12	Lorig Self-Efficacy
	LSM (95% CI)	LSM (95% CI)	LSM (95% CI)
Moderate/High Sleep Disturbance			
Yes	**1.51 (1.44, 1.59)** ***	**22.2 (21.3, 23.2)** ***	**7.00 (6.67, 7.33)** ***
No	**1.32 (1.25, 1.39)** ***	**19.8 (19.0, 20.7)** ***	**7.81 (7.51, 8.11)** ***
Age Group			
< 60	**1.35 (1.26, 1.44)** **	21.3 (20.2, 22.4)	7.32 (6.95, 7.69)
60–69	**1.42 (1.34, 1.50)** **	20.8 (19.7, 21.8)	7.46 (7.11, 7.81)
≥ 70	**1.48 (1.38, 1.58)** **	21.0 (19.8, 22.3)	7.44 (7.01, 7.86)
Sex			
Male	1.42 (1.34, 1.51)	21.0 (19.9, 22.1)	7.41 (7.05, 7.78)
Female	1.41 (1.35, 1.47)	21.1 (20.3, 21.9)	7.40 (7.13, 7.66)
Race/ethnicity			
Hispanic/Latino	1.41 (1.31, 1.52)	**22.2 (20.8, 23.6)** **	7.45 (6.98, 7.91)
Non-Hispanic/Latino White	1.43 (1.34, 1.51)	**21.4 (20.3, 22.5)** **	7.38 (7.05, 7.70)
Non-Hispanic/Latino Black	1.41 (1.33, 1.48)	**19.5 (18.5, 20.5)** **	7.39 (7.03, 7.75)
Living Below Poverty Level			
Yes	**1.49 (1.40, 1.58)** **	21.3 (20.1, 22.5)	**7.02 (6.62, 7.42)** **
No	**1.35 (1.28, 1.41)** **	20.8 (19.9, 21.6)	**7.79 (7.51, 8.07)** **
Education			
High School Grad or Less	1.44 (1.35, 1.53)	20.9 (19.7, 22.0)	7.44 (7.05, 7.82)
Some College	1.44 (1.35, 1.53)	21.4 (20.2, 22.5)	7.20 (6.82, 7.58)
College or higher	1.38 (1.29, 1.46)	20.9 (19.8, 22.0)	7.58 (7.22, 7.94)

ECOG, Everyday Cognition; LSM, least square means; CI, confidence interval.

Significant associations are boldened

* = P-value < .05

** = P-value < .01

*** = P-value < .001

## Data Availability

The datasets analyzed during the current study are available from the corresponding author upon reasonable request.
